# Supplementary material for: Uptake of Magnetite Nanoparticles on Polydopamine Films Deposited on Gold Surfaces: A Study by AFM and XPS
Source: Nanomaterials (Basel). 2024 Oct 24;14(21):1699. doi: 10.3390/nano14211699 (PMC11547837; doi:10.3390/nano14211699)

Supplementary materials

Uptake of Magnetite Nanoparticles on Polydopamine Films Deposited on Gold Surfaces: A Study by AFM and XPS

Andrea Atrei [1,*], Shalva Chokheli [1], Maddalena Corsini [1], Tóth József [2] and Giuseppe Di Florio [3]

[1] Dipartimento di Biotecnologie, Chimica e Farmacia, Università di Siena, 53100 Siena, Italy; s.chokeli@student.unisi.it (S.C.); maddalena.corsini@unisi.it (M.C.)
[2] HUN-REN Institute for Nuclear Research, H-4026 Debrecem, Hungary; toth.jozsef@atomki.hu
[3] ENEA-Italian National Agency for New Technologies, Energy and Sustainable Economic Development,
Casaccia Research Centre, 00124 Rome, Italy; giuseppe.diflorio@enea.it
* Correspondence: atrei@unisi.it

Figure S1.
Top: AFM image of the clean Au substrate. Bottom: AFM imager after immersion for 1 hour in an 1 mg/ml aqueous dispersion of magnetite NPs. After immersion the sample was thoroughly rinsed with double distilled water and dried under a flux of N_2

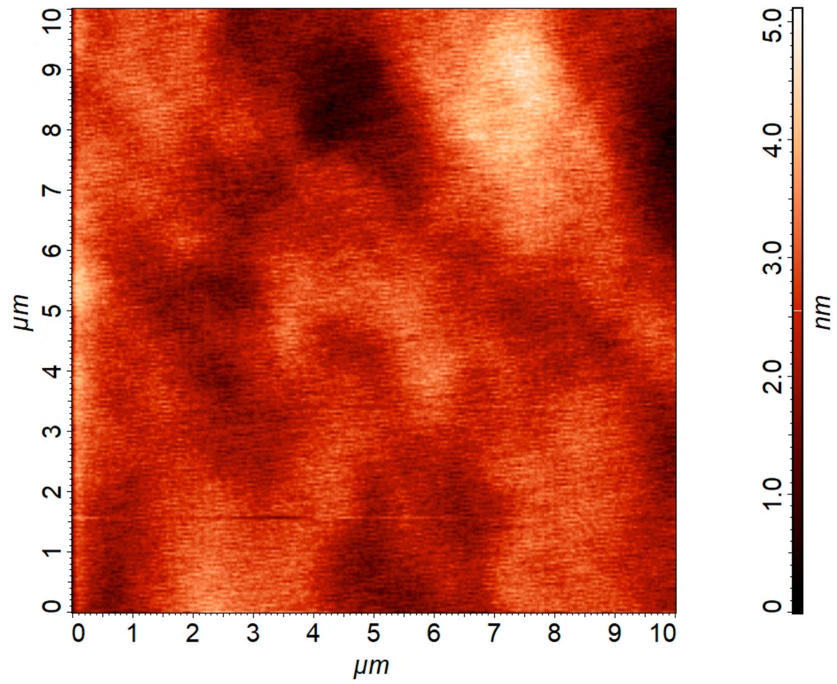

μm
0 1 2 3 4 5 6 7 8 9 10
μm
0 1 2 3 4 5 6 7 8 9 10
nm
0 1.0 2.0 3.0 4.0 5.0

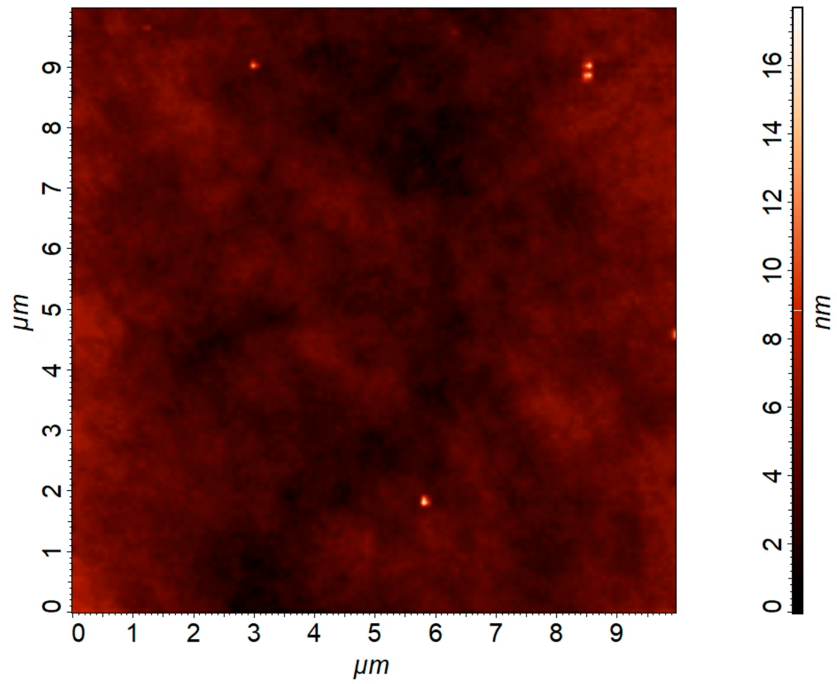

μm
0 1 2 3 4 5 6 7 8 9
μm
0 1 2 3 4 5 6 7 8 9
nm
0 2 4 6 8 10 12 14 16

Figure S2

Spectra of the Fe2p region measured for Fe_3O_4 NPs on PDA/Au films prepared with various reaction times. In the last panel the curve fitting analysis of the Fe2p spectrum of Fe_3O_4 NPs on the PDA/Au film prepared with 24 hr of reaction is shown. Fe_3O_4: Dotted curve: Experimental spectrum. Black curve: Sum of all the components. Red curve: Fe(II). Blue curve: Fe (III) in octahedral sites. Green curve: Fe(III) in tetrahedral sites. Yellow curve: Fe(II) satellite. Purple curve: Fe(III) satellite. Cyan curve: Satellite.

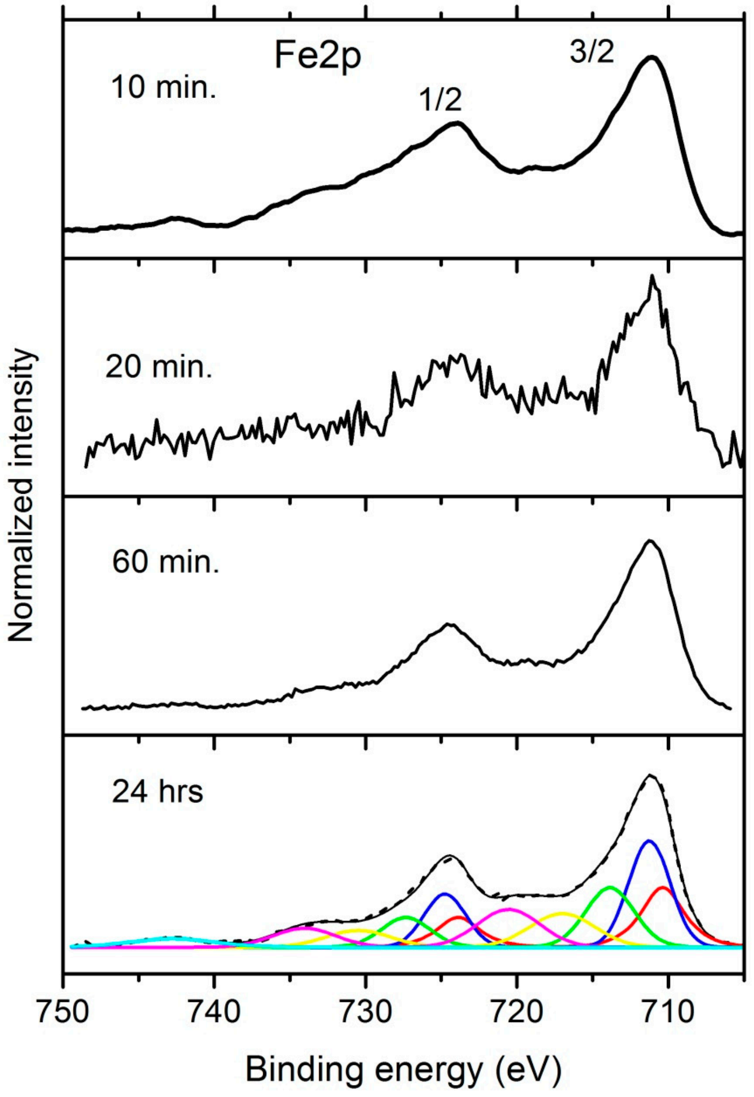

Figure S3.

C1s spectra of Fe_3O_4 NPs on PDA/Au films prepared with various reaction times. In the last panel the C1s spectra of the PDA film prepared with 24 hr of reaction is shown. The results of the curve fitting analysis of the spectra are reported. Black tick line: experimental spectrum. Black line: Sum of all the components. Red line: C-C, C-H components. Blue line: C-O, C-N components. Green curve: C=O component.

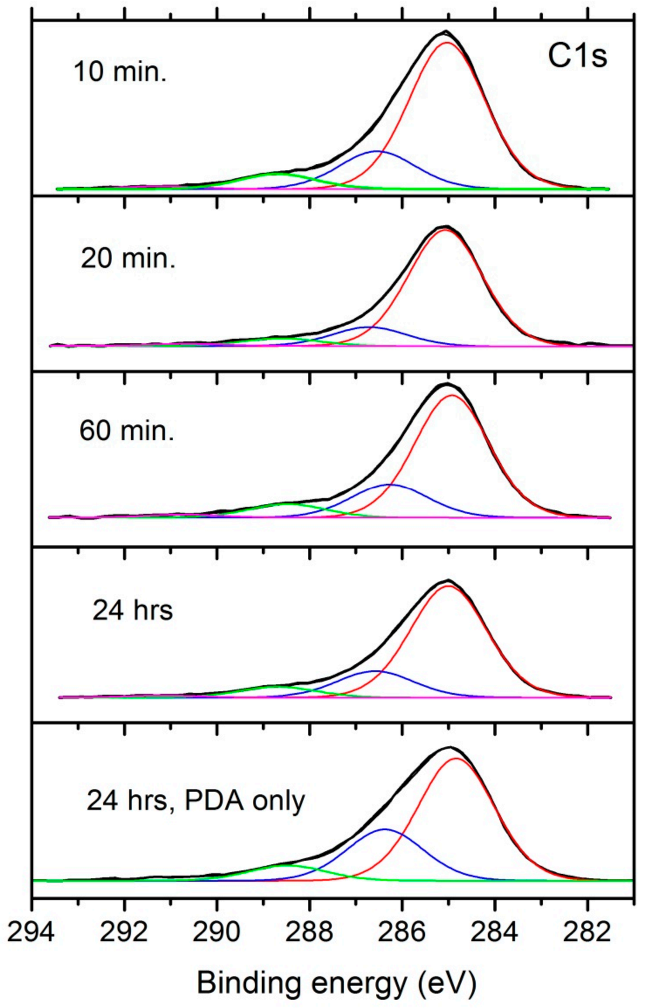

Supplement: Supplementary file 1 [file nanomaterials-14-01699-s001.zip › nanomaterials-3250075-supplementary.pdf]
